# Work related musculoskeletal stress among residents of internal medicine on cardiological wards in their daily working practice – a kinematic and ergonomic analysis

**DOI:** 10.1186/s12995-025-00471-8

**Published:** 2025-08-12

**Authors:** Fabian Holzgreve, Corinna Rittinghausen, Ingo Hermanns, Britta Weber, Rolf Ellegast, Julia Bille, Doerthe Brueggmann, Stefanie Mache, David A. Groneberg, Daniela Ohlendorf

**Affiliations:** 1 Institute of Occupational Medicine, Social Medicine and Environmental Medicine, Goethe University Frankfurt, Frankfurt am Main, 60596 Germany; 2 Institute for Occupational Safety and Health of the German Social Accident Insurance (IFA), Sankt Augustin, Germany; 3 Institute for Occupational and Maritime Medicine, University Medical Center Hamburg-Eppendorf, Hamburg, Germany

**Keywords:** CUELA, Kinematic analysis, Resident physicians of internal medicine on cardiological wards, Musculoskeletal disorder

## Abstract

**Backround:**

Earlier, interventional and sonography techniques have been analyzed in detail for the field of internal medicine concerning workplace ergonomics. Here, work related musculoskeletal disorders (WRMSDs) have been reported with a prevalence of about 76%. The aim of this study is to provide a comprehensive kinematic and ergonomic analysis of an average working day of a resident physician in internal medicine on a cardiological ward.

**Methods:**

The kinematic data of 17 resident physicians (9f/8m) of internal medicine, working in 3 different cardiological wards in a hospital of maximum care was collected on an average workday using the CUELA measurement system. A detailed, computer-based task analysis was conducted concurrently with the kinematic assessment. By synchronizing the data obtained from both measurements, postural patterns were chronologically aligned and contextualized with the corresponding task performance. The main categories were (1) Office work, (2) Ward rounds, (3) Tasks performed directly with the patient (i.e. patient examination, blood withdrawal) and (4) Other. The main categories were divided into several sub-categories for further differentiation. For the data analysis, characteristic values of joint angle distributions (percentiles P05, P25, P50, P75, and P95) for the head, neck, and torso during predefined tasks were examined and evaluated in accordance with ergonomic standards. In addition, the Owako Working Posture Analysing System was applied (OWAS).

**Results:**

A total duration of 129.2 working hours were recorded. Resident physicians of internal medicine on a cardiological ward spend a large part of their work day in office type work situations (57%) with 36% dedicated to computer work, followed by 18% for ward rounds and 16% for directly patient related activities. The office type work situations showed high sedentary rates with increased ergonomic risk for postures of the cervical and thoracolumbar spine (moderate to unfavorable postures for back curvature in almost all percentiles and office activities, reclination of the neck during (-8°- -16° in P05 and P25) for ‘use of computer’. Several aspects of patient related activities displayed high percentages of forward bending (predominately moderate and unfavorable postures for back curvature, sagittal trunk and neck inclination) and in P25-P95 for ‘blood withdrawal’ and ‘patient examination’.

**Conclusion:**

An important office type work setting is predominant in the daily routine of resident physicians of internal medicine working on cardiological wards and have been detected as important predictor to cause musculoskeletal stress. Resident physicians of internal medicine on cardiological wards have a high occurrence of ergonomically unfavorable situations, particularly during patient related activities and sedentary work using visual display units. This study highlights the need for ergonomic interventions particularly in respect to adjustable, individualized workstations and equipment.

## Introduction

Work related musculoskeletal disorders (WRMSDs) are a significant concern in healthcare settings. They can affect professionals across various specialties with high rates of musculoskeletal disorders compared to many other occupations [1–23]. Within a 12 month period 73-89.2% experienced WRMSD in at least one body region [24, 25]. Most common body regions affected are the lower back, neck and shoulders [24–26]. Due to high physical loading, several healthcare professions are considered at high risk of suffering from WRMSD. Some of these healthcare professions (nurses, orthodontists, physiotherapists and surgeons) have been looked into [9, 21, 27–32]. This includes the nursing profession, where lower back issues were the most present WRMSD with a 62% prevalence in a meta-analysis of perioperative nurses [32]. A kinematic posture analysis of orthodontists in their daily working practice revealed that awkward working postures were taken during the treatment of patients, which could cause pain and stress [28]. Physiotherapists working in a public hospital were also found to have very high rates of musculoskeletal symptoms [33]. A high prevalence of work-related musculoskeletal disorders (up to 100%) were also found among surgeons, varying for the type of surgery performed [21]. These were rarely reported and have received little attention, due to logistical restraints of studying surgical ergonomics [21].

In internal medicine, the workplace ergonomics of some specific interventionalists have been looked into. The task of sonography has also been evaluated and showed a prevalence of reported WRMSD at 76% [18, 34]. Endoscopists were found to have a 39%−89% prevalence of potential work related musculoskeletal disorders with the same tendency of under-reporting [29].

These findings show that there are several aspects within hospital and patient related work settings that merit attention in regard to the implementation of ergonomic standards.

Despite the growing interest and research on occupational health in the health care sector [1–22], there remains a gap in literature regarding the specific ergonomic challenges faced by resident physicians of internal medicine. Studies show that they spend a large majority of their time (66–81%) on indirect patient care, primarily computer-based work and documentation (38–43% of total workday) [35–39]. Direct patient contact is significantly less (13–18%). The ergonomic risks arising from these activities include prolonged static postures (prolonged sitting at computer workstations, limited breaks standing during rounds or procedures, maintaining examinations positions) and awkward positions (during patient examination) that warrant more detailed assessment and justify a study on their ergonomic well-being [40]. This is highlighted by the fact, that internal residents frequently engage in multitasking, with direct patient care often occurring simultaneously with indirect patient care activities [35].

Due to the central role of internal medicine within hospital care [41], it is of great importance to analyze this specialist discipline from an ergonomic perspective by conducting a kinematic analysis. Here, the challenge is to capture the complex work situations of a resident physician’s daily routine with diverse workflows such as office work, as well as direct and indirect patient related activities. This study considers all aspects of a resident’s daily routine. Such an analysis should provide information on the distribution and duration of activities and give detailed insights into the working postures assumed. This gives the opportunity to determine working situations with potentially harmful postures. While the data in this study was collected on three different cardiological wards, the ward work can be considered as exemplary for ward work in other disciplines of internal medicine, as it does not differ significantly.

The MAGRO-MSA [1, 9] (movement sequence analysis for medical work assessment in German hospitals) research project, of which this study is a part, recognizes that physicians health and wellbeing is not merely an individual concern but a bigger issue with implications for the quality and sustainability of health care in general. This study aims to establish a detailed and objective foundation for evidence-based workplace interventions. The goal is to improve workplace ergonomics specific to resident physicians of internal medicine on cardiological wards. A key novel aspect is not only identifying unfavorable postures but also analyzing their duration and static component. This study aims to investigate the following hypothesis:

- Hypothesis 1: Resident physicians of internal medicine working on cardiological wards spend most of their working hours doing office work.
- Hypothesis 2: Unfavorable working positions are most commonly assumed during directly patient-related activities

## Materials and methods

### Study population

Seventeen resident physicians (9f/8m) of internal medicine voluntarily participated in this study. They work in 3 different cardiological wards in a hospital of maximum care with a total of 926 in-patient beds in Germany. The average age was 31.5 years (27–39 years) and the average amount of work experience was 3.6 years. All participants had a BMI ranging from 19.4 to 26.6 kg/m² (average 23.2 kg/m²) and worked fulltime at the hospital. Exclusion criteria were known chronic disease of any kind (i.e. chronic inflammatory diseases, degenerative diseases), known musculoskeletal disorders (i.e. disc herniation, scoliosis, recent traumatic events) or BMI ≥ 30 kg/m².

They all declared to be healthy, free of any impairment and acute pain of the musculoskeletal system. In particular, no inflammatory or degenerative disease of the joints and bones had been diagnosed. They had not suffered from any severe musculoskeletal condition in the past 2 years.

This study was approved by the ethics board for research involving human subject of the Goethe University (135/14) in Frankfurt am Main, Germany.

### CUELA measurement system

Movement and posture were measured using the ambulantory measuring system CUELA (computer-assisted acquisition and long-term analysis of musculoskeletal loads). The version of the CUELA measurement system used weights about 3 kg, consists of gyroscopes and accelerometers and was developed by the institute of German occupational safety belonging to the statutory accident insurance (IFA; Sankt Augustin; Germany). The CUELA system is a wearable measurement device that is integrated into the worker’s clothing to provide objective data on body movements and postures [42]. The system is modular and includes measurement devices for the back, the lower extremities, upper extremities, neck and the head. It measures precise angles, distances and velocities of the elements being analyzed [42].

The CUELA system can deliver a detailed, objective postural analysis, including a quantification and exact duration of static postures, as well as the frequency of movements changes and the range of motion during work tasks [42, 43].

### Mini PC (objective activity analysis)

The subject was accompanied by an observer. All tasks being performed were noted using a handheld computer (UMPC, Samsung Q1, Samsung Electronics GmbH. Schwalbach, Germany), which had previously been programmed. This enables not only an attribution, but also a determination of the duration of the tasks being executed.

Before programming, interviews and observations of several residents of cardiology took place in order to determine the tasks performed on a normal working day. The main categories were (1) Office work, (2) Ward rounds, (3) Tasks performed directly with the patient (i.e. patient examination, blood withdrawal) and (4) Other. The main categories were divided into several sub-categories for further differentiation.

Subsequently the recordings of the CUELA measurement system and the data from the mini-computer were synchronized using a software (Wintervall Converter) provided by the IFA.

### Experimental procedure

The measurement took place on one randomly selected working day of a resident physician of internal medicine working on a cardiological ward. The duration of the measurement of each resident physician was approximately 2 × 4 h intervals, which corresponds to a normal day shift, excluding the lunch break. To begin with, the CUELA measurement system was attached to the test person and the belt and spinal modules were adjusted for size. It was then insured that the full range of motion was not compromised and the work clothing could be worn comfortably. Before starting the measurement, the test person was asked to stand in an anatomical, standard, neutral position, which is a future reference point for every movement performed. Then the test person was asked to perform three knee bends, a movement, which can easily be identified on the monitor afterwards. Simultaneously, the person accompanying initialized the measurement on the former programmed Mini Computer in order to synchronize the two recordings.

Throughout the day the accompanying person attributed the categories of activities mentioned above that were performed by the test person. Table 1 shows the categories and sub-categories.

**Table 1:** Presentation of main categories with respective sub-categories and their explanation

Categories	Description of sub-categories
Office (1)	
Office tasks	Sending a fax, paperwork, punching holes
Use of computer	Sitting at a desk, looking at a screen
Consulting files	Sitting or standing at a desk
Taking notes	Handwritten
Use of telephone	As solitary activity
Conversation	Mostly with colleagues, nurses
Meeting	With colleagues, i.e. to discuss radiological findings
Ward round (2)	
Consulting files	Standing at the ward trolley
Use of telephone	As solitary activity
Searching for material	Searching in the ward trolley, in cupboards
Conversation	i.e. with nurses, colleagues
Taking notes	Handwritten
Isolation measures	Putting on a surgical mask, coat, gloves etc.
Patient related tasks (3)	
Physical examination	Palpation, auscultation/inspection of thorax and abdomen, and of lower extremities
Ultrasound	Mainly of thorax and abdomen, rarely color duplex sonography of the lower extremities
Puncture	Pleural drainage or ascites drainage, bedside
Connection/disconnection of intravenous drip	Mainly to the patient’s arm, bedside
Blood withdrawal	Mostly bedside from the patient’s arm or hand
Bedside Conversation	Often sitting or bending down to patient
Resuscitation measures	Cardiac massage, bedside
Defibrillation	Bedside emergency defibrillation or planned cardioversion
Other (4)	
Hygiene	Washing/disinfection of hands
Waiting	
Walking	Covering distances, climbing stairs
Non job related activity	Excluded from assessment
Taking a break	Excluded from assessment
Evaluating blood gas analysis	Standing in front of the BGA machine

Instrumental examinations specific to the discipline of cardiology, such as cardiac catheterization for example, were not part of the evaluation.

### Data evaluation

The data was synchronized using the WIDAAN software developed for the CUELA system.

The range of angles for each body area recorded by the CUELA system (head flexion/extension, neck curvature and inclination to the side; trunk inclination to the front and side, back curvature to the front and back torsion) were then color coded. This demonstrates their association with neutral, moderate or unfavorable working postures in accordance with ergonomic standards (Tables 2 and 3), their potential threat to the musculoskeletal system and the necessity to intervene [44, 45].

**Table 2:**
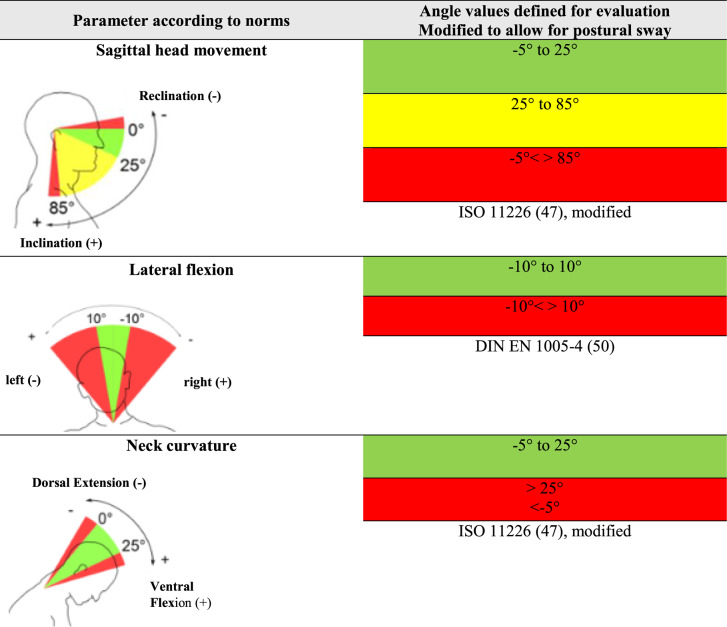
Joints or body regions of the head and neck, and their corresponding degrees of freedom, measured by the CUELA system. It also shows the color coded angle values for each joint/body region, which illustrates the distinction between neutral, moderate and awkward postures, based on ISO 11,226 and DIN En 1005−4, modified to allow for postural sway [44, 46–48]

**Table 3:**
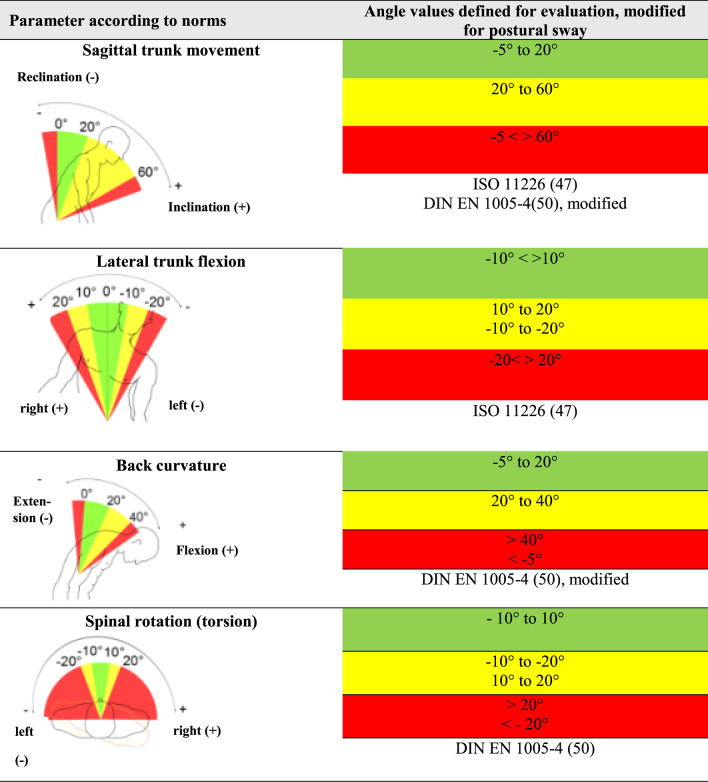
Shows joints or body regions of the trunk, and their corresponding degrees of freedom, measured by the CUELA system. It also shows the color-coded angle values for each joint/body region, which illustrates the distinction between neutral, moderate and awkward postures, based on ISO 11,226 and DIN En 1005−4, modified to allow for postural sway [44, 46–48]

To allow for postural sway, 5° tolerance was added to the neutral position for the head flexion/extension, neck curvature, trunk inclination and back curvature.

The percentiles P05-P95 were examined, thus eliminating data, which is not representative of the normal distribution of results. The percentiles indicate the value below which a given percentage of observations in a group of observations falls. In this case for example, the 5th percentile (P05) describes the body angle for a given activity throughout the measurement duration that 5% falls below. Ergo the 95th percentile (P95) describes the body angle for a given activity throughout the measurement duration that 95% fall below.

To describe the variance of the test person’s movements within one activity, the modified interquartile range (mIR= [(P50-P05]+[P95-P50]/2) was calculated. It is determined by the average difference between the 5th and 50th as well as between the 50th and 95th percentiles. The greater the value, the greater the motion variance will be. The mIR was first used by Hauck et al. [45] to give more precise insight into the descriptive data in addition to the percentiles. It allows a more precise interpretation of the data with regard to motion variance, explaining the extent to which the respective activity can be carried out.

Additionally, the OWAS assessment method was conducted, which was developed as an observational method. In this case the evaluation was performed by a software, using the recorded body angles by the CUELA system.

The evaluation parameters specify the exact angle values for a particular body region. If a rotation, curvature or inclination is described in one direction (positive sign), the negative sign of the value refers to the opposite direction of the movement [45]. This classification used follows the standardization [44].

## Results

A total duration of 129.2 working hours were recorded. Resident physicians of internal medicine working on cardiological wards spent 57% (74.5 h) of their working hours doing office work, of which the majority of time was spent in front of a computer (63%, 45.5 h).

Ward rounds took up 18% of their time and 16% were spent doing patient-related activities.

The large majority of the category “other” consisted of walking with a total of 10 h and 40 min. Non-job related activities and small breaks took up an additional 60 min of the total recording time in the category “other” and were excluded from evaluation.

The following results concentrate on the activities and postures with high occurrence of moderate and unfavorable angle ranges of each category.

In the category ‘office’ the sagittal head inclination during the activity ‘consulting files’ showed P50-P95 values in a moderate range (31–47°, mIR 19°). ‘Office tasks’ were performed in a moderate range at P75-P95 (37°−49°, mIR 22°).

For the neck curvature to the front the P25 values for the activities ‘conversation’ (−10°) and ‘use of computer’ (−8°) fell into the unfavorable range.

The angles for the back curvature to the front all lie in the moderate to unfavorable range (23°−55°, mIR 11°−16°) with the exception of the P05 value for the activity ‘office tasks’ (15°). Particularly the activity ‘conversation’ stands out with P50-P95 values all in the unfavorable range (42°−55°). Table 4 shows the angle values of the category office.

**Table 4:**
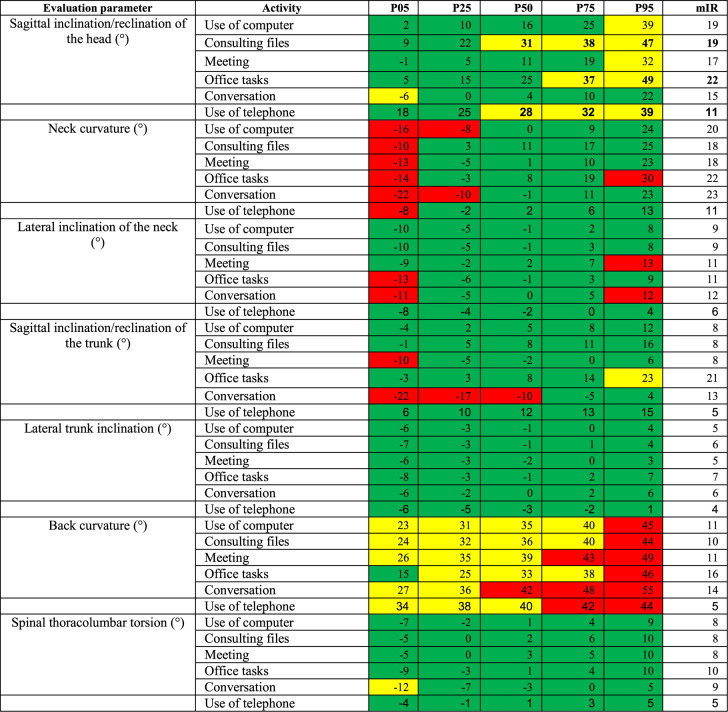
Percentiles P05, P25, P50, P75, P95 and the mIR for the category 1 ‘office’

The results for the category ‘ward round’ show a neutral to moderate value range for the head tilted to the front (−4°−58°; mIR 12°−27°). The P25-P95 values for the activities ‘taking notes’ and ‘waiting’ were all in the moderate range, as were the P50-P95 values for the activities ‘consulting files’ and ‘searching for material’.

For the neck curvature to the front the activity ‘waiting’ showed unfavorable ranges for all percentiles (34°−40°, mIR 10°) with the exception of P05. The mIR for the neck curvature in this category varied between 10°−21°.

The activity ‘waiting’ had neutral P75-P95 values for lateral neck inclination, but showed unfavorable values for P05-P50 (−15° to −13°, mIR 11°) and a clearly dominant inclination to the left (represented by the negative prefix). The mIR for the sideway neck inclination in this category varied between 9° and 12°.

The forward inclination of the trunk presents P75-P95 values for the activity ‘searching for material’ (32°−46°), which lie in the moderate range. The mIR lies between 3°−21° for the forward inclination of the trunk in this category.

The back curvature to the front displays P95 values for the activities ‘taking notes’ (24°) and ‘consulting files’ (22°), which were in the moderate range. The P75-P95 values of the activity ‘searching for material’ (29°−41°) were in the moderate, respectively unfavorable range. The mIR here varied from 3°19°.

These findings are illustrated in Table 5.

**Table 5:**
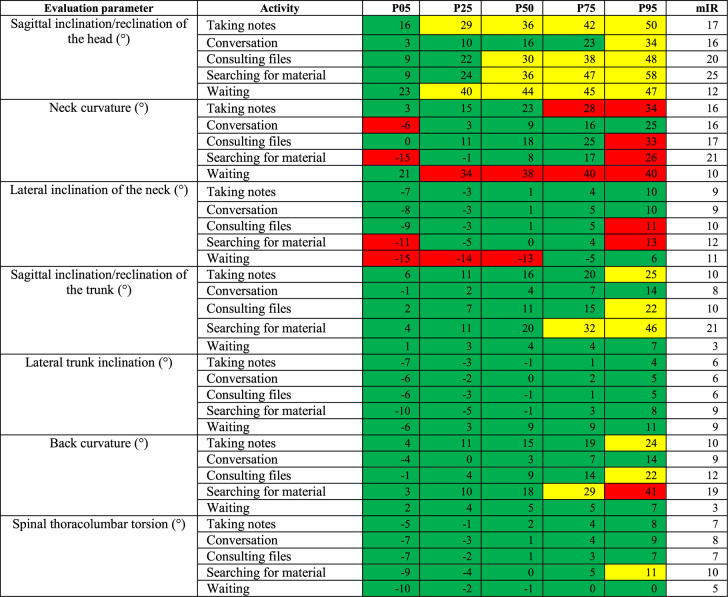
Percentiles P05, P25, P50, P75, P95 and the mIR for the category 2 ‘ward round’

In the category ‘patient related tasks’ moderate values from P25-P95 were found for the head tilt to the front for the activities ‘blood withdrawal’ and ‘physical examination’ (30°−67°, mIR 21°−25°). The activity ‘bedside conversation’ showed P75-P95 values in the moderate range (28°−40°, mIR 18°).

The activities ‘blood withdrawal’ and ‘ultrasound’ revealed unfavorable values for P05-P50 (−29° to −6°) for the neck curvature, so did the activity ‘bedside conversation’ for P05 and P95 (−8°−26°). The mIR for the neck curvature to the front varied from 17°−23°.

The trunk inclination to the front displayed moderate angle values for P25-P95 for the activities ‘blood withdrawal’ and ‘physical examination’ (21°−54°), as well as the P95 value for the activity ‘ultrasound’. The mIR varied from 9°−23°.

For the back curvature to the front only the P05 value for the activity ‘blood withdrawal’ lay in the neutral range (11°). The P25 value lay in the moderate range (30°). The P50-95 values lay in the unfavorable range (41°−56°). The mIR here was 29°. The activity ‘physical examination’ showed neutral angle values for P05-P25 (6°−20°) and moderate angle values for P50-P75 (30°−38°). The P95 value lay in the unfavorable range (45°) with an mIR of 20°. For the activity ‘ultrasound’ angle values for all percentiles lay in the moderate or unfavorable range (P05-P50: 21°−40°; P75-P95: 45–52°, mIR 16°).

There was a slight deviation to the right for back torsion for the activities ‘blood withdrawal’ (−9° vs. 14°) and ‘patient examination’ (−7° vs. 13°), which means that the test persons were leaning more towards the right. Table 6 illustrates these findings.

**Table 6:**
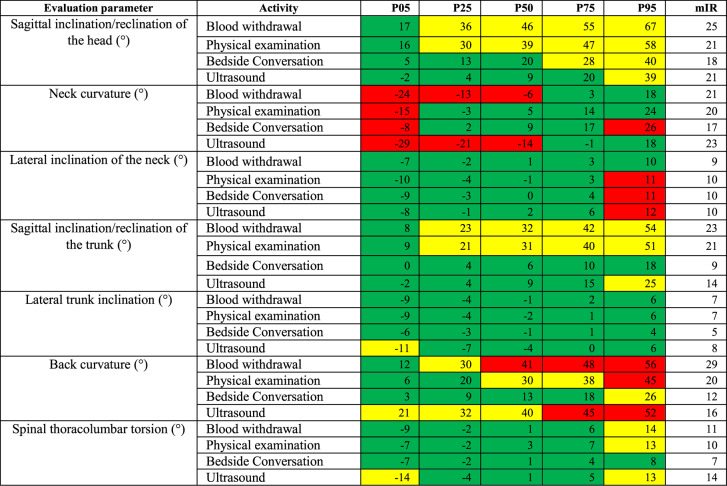
Percentiles P05, P25, P50, P75, P95 and the mIR for the category 3 ‘patient related tasks’

## Discussion

For the first time, the entire workday of resident physicians of internal medicine on cardiological wards has been examined, revealing precise information on postures adopted during different activities throughout the workday. This allows a detailed kinematic posture and ergonomic analysis.

Residents of internal medicine on cardiological wards spent 57% of their total working time doing office work (category 1), of which the majority of time was spent in a sitting position, working with visual display units (63%). This amounts to 36% of total working time, highlighting the importance of sedentary computer-based work in this line of profession. These findings confirm hypothesis 1, which is therefore accepted. This confirms the work of Wieler et al. [36] who found that year one residents of internal medicine spent 38% of their time doing computer based work. Chaiyachati et al. [35] differentiated between direct and indirect patient care and found that interacting with the medical record, which mainly involved documentation and working with electronic health records, was the dominant activity in indirect patient care, accounting for 43% of the day. This again correlates our findings and highlights the relevance of sedentary work for internal medicine resident physicians on cardiological wards.

Another study finding similar time allocations for office work for neurologists in a hospital ward setting, was that of Bijanzadeh et al. [1], which had a similar study design to this study as it also belongs to the MAGRO-MSA project [1, 9].

The evaluation of the percentiles display that strenuous positions are assumed during a normal working day of resident physicians of internal medicine in cardiology. The focus of the body areas vary, depending on the different categories and activities. In the category ‘office’ the main concern is the back curvature to the front, which revealed moderate to unfavorable values for almost the entire working time and activities.

This could be due to slumped sitting and insufficient support of the lumbar spine during seated position. Also, a lack of individual adaption of the chair could contribute to this negatively. Sedentary work has a high impact on back posture and back pain. Brakenridge et al. [49] looked into the relationship between sitting reduction and musculoskeletal pain, finding that reductions in sitting time were significantly associated with reduced lower back pain. Akkarakittichoke et al. [50] found that office workers who received interventions to reduce sitting discomfort through active breaks and postural shifts experienced significantly shorter recovery times from low back pain and lower recurrence rates compares to control groups.

The sagittal backward neck curvature during office work could be due to a worker-workstation mismatch. If the computer screen is placed to high for example, an unfavorable, static backward bending of the cervical spine results. Adjusting the monitor height so that the top of the monitor is at eye level or slightly below helps maintain a neutral head position [51].

Previous research has shown that sedentary office work significantly impacts neck posture and pain. Andersen et al. [52] found that neck and shoulder complaints are common among employees in sedentary occupations characterized by intensive computer use. Their research established that specific strength training can be a promising intervention for relieving neck pain in office workers. Lee et al. [51] showed that ergonomic work station adjustments reduced neck pain intensity of office workers, compared to a control group. They found that interventions addressing workstation mismatches based on individual measurements were effective in reducing neck pain over a 36-week period.

The categories 2 and 3 (ward rounds and patient-related activities) were evenly distributed. Ward rounds took up 18% and patient related tasks 16% of the overall time. These findings correlate with the findings from other studies, where direct patient related activities were found to take up 13–18% of the time [35, 36] and ward rounds took up 12.5% of the time [35].

In the category ‘ward round’, the head and cervical spine, displayed the highest occurrence of moderate and unfavorable angle values. Particularly the activities ‘consulting files’ and ‘taking notes’, showed P25-P95 values for the activities ‘taking notes’ and P50-P95 values for the activities ‘consulting files’ in the moderate range of head inclination. These two activities together took up 56% of the time in this category. A high percentage of these awkward postures were detected (74.7% for taking notes, and 59.7% for consulting files). The neck curvature also showed a high percentage of awkward postures in these two activities suggesting a ‘looking down’ movement of the cervical spine dominated these activities. This is possibly due to non-adjustable equipment.

The category ‘patient related tasks’ also showed a high rate of moderate and unfavorable angle values for the head and cervical spine as well as for the trunk inclination and back curvature. This was particularly the case for the activities ‘blood withdrawal’ and ‘patient examination’. These activities showed a high occurrence of back curvature, neck curvature, neck inclination and trunk inclination in moderate to unfavorable ranges, suggesting a frequent forward bending movement during these tasks. This confirms hypothesis 2.

These findings could be the consequence of not adjusting working materials (i.e. bed height) and further emphasizes the need for intervention.

For the activity ‘ultrasound’ unfavorable postures were documented in particular for the neck (unfavorable angle values in P75-P95) and back curvature (unfavorable angle values in P05 to P50), as well as the sagittal trunk inclination (moderate angle values for P25-P95). The unfavorable sagittal neck movement was in the − 14° to −29° range and represents a backward bending of the cervical spine, as when looking up. In analogy to the office setting, this indicates a mismatch between the height of the ultrasound screen and the residents’ eyes.

Category 4 (‘other’) consisted largely of the activity ‘walking’, with body postures predominantly in the neutral range. It was not further investigated as the focus of this study lies on identifying potentially hazardous ergonomic workplace situations. It would be assumed that walking, on the contrary, offers a postural variety in the daily routine that could be considered a beneficial factor in this context [50].

Further investigations, weighing such contributing factors, would be necessary in the future.

In the initial interpretation of the results, the extension of the head and entire spine showed high percentages of unfavorable positions (traffic light color code: red) in all categories. Looking more closely at the results, these unfavorable positions were mostly slight deviations from the norm, within the − 5° to 0° range of motion and represented the smallest of movements, caused for example by breathing. Since the ergonomic standards refer to a person who is standing still and are based mainly on observations and not on precise angle measurements, a deviation of the extension of the head, cervical spine, thoracic spine or trunk in this area might indeed be unfavorable. However, for a person in motion these minimal deviations from the neutral posture can be attributed to postural sway and were thus interpreted. Evaluating these findings, it was assumed that the ergonomic standards in combination with the exact determination of body angles may be too strict and do not represent the situation realistically in this very small angle range.

This is why in this study the angle range for the extension of the head, cervical spine, thoracic spine and trunk was modified from ergonomic standards for assessment by the CUELA system to allow for postural sway and prevent a deceptive interpretation of the results. The neutral position was extended from 0° to −5° for the above mentioned body angles. The outcome that a 4° or 6° deviation from the neutral position would have had on our results was not tested, probably slightly less or respectively more unfavorable positions would be the outcome. Further research in this specific area would be necessary to show which exact angle deviation from neutral is most representative in a real life situation and includes postural sway without overlooking actual unfavorable positions.

Looking at the percentiles (P05-P95) gives a more accurate impression, as such ‘extreme’ findings are put into perspective by concentrating on the representative proportion of the measurement.

The CUELA measurement system is complex, particularly concerning the large amount of data collected, which requires careful analysis. The measurement devices must be fitted to the test person with great care and the correct calibration requires some experience in order to avoid error in the collected data. The Hawthorne effect may have an impact on the expected results, however, the long duration of the measurement intervals makes relevant, lasting alterations in posture unlikely and the purely descriptive nature of the observation limits the possible distortion on this side.

The focus of this study lies on postures of the head and trunk, while finger movements, i.e. of the hand/wrist region were not captured in this study and could be investigated in the future.

The aspect of repetition of awkward movements or postures, though relevant to the occurrence of MSDs [53], was not a part of this study.

Some aspects of the workload of resident physicians of internal medicine on cardiological wards, that could contribute to the risk of developing MSDs, were not considered in this study. Forces caused by external factors, as for example pushing and pulling of heavy ward round carts, ultrasound machines, patient beds or handling of heavy patients during examinations and procedures could potentially also affect the occurrence of MSDs in this context and should be included in future studies [54].

This study emphasizes the need for ergonomical workplace design. In this study population in particular, structural preventions measures such as height adjustable monitors could contribute largely to an improvement of head/neck posture during office work and ultrasound examinations, as well as height adjustable ward trolleys could during ward rounds. Behavioral preventive measures such as strength training could help counteract muscle weakness and postural imbalances caused for example by prolonged sitting. Raising awareness for ergonomic workplace issues through preventive education also seems highly relevant. Though adjustable chairs were mostly available, as were height adjustable patient beds, they were often not made use of. A more heedful use of these aids could surely lead to an improvement of back posture for this professional group.

## Conclusion

Resident physicians of internal medicine on cardiological wards spend a large part of their workday in office type work situations (57%) with 36% dedicated to computer work, followed by 18% for ward rounds and 16% for directly patient related activities. The category office work is mainly sedentary, which involves prolonged, ergonomically unfavorable postures of the cervical and thoracolumbar spine that may contribute to MSDs. The main aspect of the cervical spine in the office type setting was a backward bent position during computer-based work the percentiles showing unfavorable postures for P05-P25. During directly patient related activities, particularly ‘patient examination’, ‘blood withdrawal’ and ultrasound, high occurrence of extreme postures (color code red) was described. This kinematic analysis shows, that there is great need for ergonomic workplace interventions, particularly regarding the sedentary aspect of this workplace as well as implementing adjustable mechanical aids for direct patient tasks, when needed.

## Data Availability

No datasets were generated or analysed during the current study.
